# Rat models of acute inflammation: a randomized controlled study on the effects of homeopathic remedies

**DOI:** 10.1186/1472-6882-7-1

**Published:** 2007-01-17

**Authors:** Anita Conforti, Paolo Bellavite, Simone Bertani, Flavia Chiarotti, Francesca Menniti-Ippolito, Roberto Raschetti

**Affiliations:** 1 Department of Medicine-Public Health, University of Verona, Policlinico G.B. Rossi, Piazzale L.A. Scuro, 33100 Verona, Italy; 2 Department of Morphological-Biomedical Sciences, University of Verona, Policlinico G.B. Rossi, Piazzale L.A. Scuro, 33100 Verona, Italy; 3 Department of Cell Biology and Neuroscience, Istituto Superiore di Sanità, Viale Regina Elena 299, 00161 Rome, Italy; 4 National Centre for Epidemiology, Surveillance and Health Promotion, Istituto Superiore di Sanità, Viale Regina Elena 299, 00161 Rome, Italy

## Abstract

**Background:**

One of the cardinal principles of homeopathy is the "law of similarities", according to which patients can be treated by administering substances which, when tested in healthy subjects, cause symptoms that are similar to those presented by the patients themselves. Over the last few years, there has been an increase in the number of pre-clinical (*in vitro *and animal) studies aimed at evaluating the pharmacological activity or efficacy of some homeopathic remedies under potentially reproducible conditions. However, in addition to some contradictory results, these studies have also highlighted a series of methodological difficulties.

The present study was designed to explore the possibility to test in a controlled way the effects of homeopathic remedies on two known experimental models of acute inflammation in the rat. To this aim, the study considered six different remedies indicated by homeopathic practice for this type of symptom in two experimental edema models (carrageenan- and autologous blood-induced edema), using two treatment administration routes (sub-plantar injection and oral administration).

**Methods:**

In a first phase, the different remedies were tested in the four experimental conditions, following a single-blind (measurement) procedure. In a second phase, some of the remedies (in the same and in different dilutions) were tested by oral administration in the carrageenan-induced edema, under double-blind (treatment administration and measurement) and fully randomized conditions. Seven-hundred-twenty male Sprague Dawley rats weighing 170–180 g were used. Six homeopathic remedies (*Arnica montana D4*, *Apis mellifica D4*, *D30*, *Atropa belladonna D4*, *Hamamelis virginiana D4*, *Lachesis D6*, *D30*, *Phosphorus D6*, *D30*), saline and indomethacin were tested. Edema was measured using a water-based plethysmometer, before and at different times after edema induction. Data were analyzed by ANOVA and Student t test.

**Results:**

In the first phase of experiments, some statistically significant effects of homeopathic remedies (*Apis*, *Lachesis *and *Phosporus*) were observed (the reduction in paw volume increase ranging from 10% to 28% at different times since edema induction). In the second phase of experiments, the effects of homeopathic remedies were not confirmed. On the contrary, the unblinded standard allopathic drug indomethacin exhibited its anti-inflammatory effect in both experimental phases (the reduction in paw volume increase ranging from 14% to 40% in the first phase, and from 18% to 38% in the second phase of experiments).

**Conclusion:**

The discrepancies between single-blind and double-blind methods in animal pharmacological research are noteworthy and should be better investigated, also in non-homeopathic research.

## Background

One of the cardinal principles of homeopathic theory of medicine is the "law of similarities", according to which patients can be treated by administering substances which, when tested in healthy subjects, cause symptoms that are similar to those presented by the patients themselves. Another important principle is that of "minimal dilution": to use the lowest concentration of a substance that still provokes a response [1].

Over the last few years, there has been an increase in the number of pre-clinical (*in vitro *and animal) studies aimed at evaluating the pharmacological activity or efficacy of some homeopathic remedies under potentially reproducible conditions. Among them, animal studies have shown that a homeopathic complex containing low dilutions/dynamizations of *Arnica montana*, *Atropa belladonna*, *Hamamelis virginiana *and other compounds has a slight but significant effect on experimental rat paw inflammation caused by the injection of autologous blood [2] and during the acute phase of arthritis induced in rat by means of the injection of heat-killed *Mycobacterium butyricum *[3]. Moreover, *Apis mellifica *is a hydro-alcoholic extract of the body of bee that, according to the law of similarities and data derived by homeopathic literature, could have anti-inflammatory or anti-edemagenic activity [4-8]. *Lachesis*, the venom of the *Crotalus mutus *snake, and *Phosphorus *are remedies indicated in the homeopathic literature for diseases with hemorrhagic tendencies, but no controlled investigations have been published so far. High dilutions/dynamizations of *Atropa belladonna *[9] and *Phosphorus *[10] have been found to have a slight *in vitro *inhibitory effect on neutrophil granulocytes, which play a fundamental role in acute inflammation. However, in addition to some contradictory results, the pre-clinical studies have also highlighted a series of methodological difficulties, related to the very low concentration and activity of the medicines (requiring higher number of experimental animals) and to the largely unknown factors affecting their stability in time or their pharmacokinetics [11]. On the other hand, small clinical studies, carried out under selected conditions on *Arnica montana*, *Atropa belladonna *and *Hamamelis virginiana*, have found that, alone or in combination, they could have some anti-inflammatory activity [12-17].

Therefore, the objective of our study was to evaluate, through an animal-based model, the efficacy of the above-mentioned remedies, which are usually prescribed by homeopathists to treat clinical conditions characterized by inflammatory or hemorrhagic manifestations.

The study was organized in two distinct randomized experimental controlled phases.

Experiment A was planned to study the largest possible number of conditions (homeopathic remedies, administration routes and edema models) compatible with the technical requirements and reasonable costs of the tests. Subsequently, a selection of the above-mentioned conditions was re-tested (experiment B) with special attention to confounding factors, which could interfere with the assessment of treatments. Specifically, we re-tested those homeopathic remedies, edema model and administration route which in Experiment A gave paw volume increases significantly lower than physiological solution, at most times since edema induction.

This study was conducted within a National Project on Unconventional Therapies coordinated by the Italian National Institute of Health (Istituto Superiore di Sanità, Rome, Italy) and funded by the Italian Ministry of Health.

## Methods

The general scheme of the study is shown in Table 1. On the overall, 40 different experimental treatment groups were studied, namely 32 groups in Experiment A (2 edema models × 2 administration routes × 8 treatments) and 8 groups in Experiment B (1 edema model × 1 administration route × 8 treatments).

**Table 1:** General scheme of the study

			**Phase A**		**Phase B**
**Randomisation**					
by cages					
within cages by treatments			-		

**Blinding**					
Treatment administration			-		
Edema measurement					

**Inflammation model**			**CA**	**BL**	**CA**

**Homeopathic remedies**					
	dilution	n. of animals studied			
				
Arnica	D4	71	O/I	O/I	-
Apis	D4	90	O/I	O/I	O
Apis	D30	18	-	-	O
Atropa	D4	72	O/I	O/I	-
Hamamelis	D4	72	O/I	O/I	-
Lachesis	D6	86	O/I	O/I	O
Lachesis	D30	18	-	-	O
Phosphorus	D6	90	O/I	O/I	O
Phosphorus	D30	18	-	-	O
					
**Reference treatments**					
Placebo:					
Physiological solution		86	O/I	O/I	O
Active comparator:					
Indomethacin		88	I/I	I/I	I

**O **= oral administration

**I **= administration through injection

**CA **= carrageenan induced edema

**BL **= autologous blood induced edema

Each treatment group has been tested through 3 replications. In each replication 6 rats were studied. In experiment A 11 rats were excluded from the analyses because of problems with edema induction.

### Study setting

All the experimental activities were carried out at the Departments of Medicine-Public Health and of Morphological-Biomedical Sciences, University of Verona. Data analysis was performed at the Istituto Superiore di Sanità in Rome.

### Animals

Male Sprague Dawley rats (Harlan Italy) weighing 170–180 g were used for both experiments A and B. Rats were housed for six days from arrival to testing, and their individual weight was collected at arrival and at the day of testing. Increase in rat weight was used as a measure of animal well being. During the course of all replications (experiments A and B), the animals were kept in a room other than that used for the treatments and measurements.

The study was conducted in conformity with the Italian regulations governing the protection of laboratory animals used for experimental purposes (permission granted by Ministry of Health, according to Law Decree No. 116/92). At the end of each experiment, the animals were sacrificed by means of ether anesthesia.

### Remedies

We chose to use the homeopathic remedies *Arnica montana*, *Atropa belladonna*, *Apis mellifica*, *Hamamelis virginiana*, *Lachesis*, and *Phosphorus *at the lowest marketed dilutions/dynamizations (from D4 to D6, depending on the remedy). This choice was based on the hypothesis frequently sustained in the homeopathic literature that the treatment of local symptoms and acute cell or organic reactions requires lower doses than the treatment of general symptoms and chronic diseases. The D30 dilution was added in the second phase of experiments, to compare possible dose-related differences in treatment effects. Furthermore, we chose to use the preparations available in physiological solution because this allowed us to administer the same formulation orally and by injection. The two ways of administration were initially chosen because this experiment was designed to explore and screen the widest possible range of methodological variables, in order to maximize the possibility of pointing out significant effects, if present. The homeopathic remedies (1 ml glass vials containing a sterile isotonic solution) were produced in accordance with the German homeopathic pharmacopoeia (HAB) [18]. Sterilization was done by dry heat sterilization, ionizing radiation, or filtration according to the different substances of the mother tincture, as described in HAB (Arnica rule n. 4a, Atropa belladonna rule n. 2a, Hamamelis rule 3a, Apis mellifica rule 4b, Lachesis e Phosphorus follow specific rules). Dilution and dynamization (succussion) was done in glass vials under sterile conditions.

A sterile saline physiological solution (0.9% NaCl) was used as reference inert treatment (placebo). Indomethacin (Sigma) was dissolved in sterile water for injections at a concentration of 10 mg/ml immediately before the tests. Indomethacin was administered intramuscularly as active reference treatment (standard non-steroidal anti-inflammatory drug), to check the suitability of the model in revealing a potential drug effect, if present. Therefore, we were not interested in comparing quantitatively its effect with those of homeopathic medicines.

### Animal models

We used two different rat models of acute inflammation, in order to explore the effect of a series of treatments in conditions which differed, at least in part, in their pathophysiological mechanisms. Specifically, the first model is based on the use of a classical irritating, edema-causing, substance (carrageenan-induced edema), and involves the activation of the arachidonic acid cascade, giving rise to the formation of the principal mediators of inflammation (prostaglandins and thromboxanes). This model is commonly used to screen conventional non-steroidal anti-inflammatory drug [19]. The second one (used only in experiment A) is the autologous blood-induced edema model recently developed by the Verona group, which mimics a traumatic condition involving the perfusion of blood into the joint (typical of common sprains and bruises) and the development of inflammation lasting a few hours. We already used it to study the regulating power of a homeopathic complex [2].

### Edema induction

The edema was induced by injecting 0.1 ml of carrageenan 0.5% in physiological solution or 0.1 ml of autologous blood into the sole of the right posterior paw. The carrageenan (Sigma) was dissolved the day before the experiment, homogenized in a potter and stored in the dark at +4°C. Preliminary studies have shown that this dose of carrageenan induces a medium-large edema in comparison with a maximum dose. Immediately before the experiment, the solution was re-homogenized. The autologous blood was drawn from two ether-anesthetized syngenic rats by means of a cardiac puncture, and made uncoagulable by the addition of heparin.

### Treatments

The homeopathic remedies were administered immediately after edema induction. The oral treatment was performed using an insulin syringe without a needle spraying 0.1 ml of the remedy or of the physiological solution (control group) into the oral cavity above the tongue, and then immediately returning the animal to its cage. The injection treatment was performed injecting 0.1 ml of the remedy or of physiological solution into underside of the right posterior paw using an insulin syringe. Indomethacin (standard treatment) was injected intramuscularly (0.1 ml/hg) 30 minutes before edema induction in order to allow its absorption. In the sub plantar experiments, the group of rats treated with indomethacin was also given a sub plantar injection of 0.1 ml of sterile physiological solution at the time of edema induction. In this way, the initial increase in paw volume due only to the sub plantar injections (a total of 0.2 ml) was the same as that in the rats treated with the homeopathic remedies.

### Treatment assignment

At arrival, animals were taken from transport cages one-by-one and sequentially transferred to the homecages. In Experiment A the subsequent couples of animals were located in different cages, until six animals were housed in the same cage. The whole cage was then assigned to one of the different treatments, according to an assignment list different from replication to replication. On the contrary, in Experiment B eight animals consecutively taken from transport cages were housed in the same homecage, and then assigned to treatments (one animal per treatment) according to an assignment list different among cages and replications.

### Blinding procedures

Experiment A. Homeopathic remedies were sucked up by a syringe directly from the purchased glass vials just before administration to animals. All cagemates received the same treatment, which differed from cage to cage. Cages were attributed different codes, and animals within cages were individually marked with a permanent staining on different parts of the body. The person charged with paw volume measurements was the only one that was blind to treatments among those working on animals (single blind). He used cage and animal codes to recognize individuals and to report repeated measurements on data collection forms. Experiment B. The homeopathic remedies and the sterile physiological solution of 0.9% NaCl were transferred (1 ml) in 1.5 ml sterile plastic vials with caps and labeled at the Department of Morphological-Biomedical Sciences, University of Verona. These vials were then sent to Istituto Superiore di Sanità (ISS), Rome, in a single package box divided in several compartments, where each vial was separated from the others by a cardboard wall. At ISS, a person not involved in the experimental trial coded them with a letter, and sent them back to the experimental laboratory (Verona), where they were used within 1 month. Animals were housed in 6 cages (8 rats in each cage) and individually marked with a permanent staining on different parts of the body. Each cagemate received a different coded treatment. The correspondence between code and remedies was notified only at the end of the experiments and after the statistical analyses were completed. Therefore, treatment administration and paw volume measurement were performed by persons both blind to treatments (double-blind). Blinding was not applied to indomethacin treatment.

After coding of treatments, a qualitative analysis was performed through ultraviolet (UV) absorption spectra on samples of homeopathic treatments, saline and indomethacin. UV analysis performed on homeopathic and saline samples showed the absence of the characteristic absorption band of indomethacin (maximum at 318 nm, according to the European Pharmacopoeia).

### Measurements of the edema

The paw volume was measured, in both experiments A and B, using a water-based plethysmometer (U. Basile, Milan) before edema induction (time 0) and after 1, 3, 5 and 7 hours (carrageenan-induced edema) or 1, 2, 3 and 5 hours (blood-induced edema). The change in paw volume due to the carrageenan or blood injection, edema and eventually remedy (in the case of sub plantar route of administration), was computed for each rat as difference between paw volume at each time from edema induction (1 hr, 2 hr, 3 hr, 5 hr, 7 hr) and paw volume immediately before edema induction (time 0 = baseline). In the following, such transformed data are called differential paw volume data.

Finally, one non-treated rat was repeatedly tested throughout experiment B, to estimate the reproducibility of the measurement instrument.

### Statistical analysis

Sample size was estimated considering the two-tailed Student t test for independent groups performed to test efficacy. Specifically, we based our calculation on the following values for the different parameters: (i) standard deviation of paw volume increase homogeneous among groups σ = 0.10 (based on control group data from previous works); (ii) the smallest difference in paw volume increase between treatment and saline, worth detecting from a biological/clinical point of view, Δ = 0.1275 (i.e. Δ = 1.255σ, corresponding to the half-width of the 80% reference interval of control animals); (iii) Type I error probability α = 0.007 (corresponding to an experimentwise probability α_E _= 0.05 when considering the correction for seven comparisons); (iv) power 1-β = 0.80. The resulting sample size per group was n = 18.

The following statistical analyses were performed on collected data. Differential paw volume data were checked for normality, using the Shapiro-Wilks test. At all times data respected the normality assumption. Moreover, the Levene test, performed to assess the homogeneity of variance between groups, did not show any significant difference. Therefore, the use of parametric test for the assessment of treatment effect was justified, as expected.

A mixed model analysis of variance (ANOVA) was then performed on differential paw volume data. When analyzing the repeated measures, the Huynh-Feldt correction was used to take into account possible violation of the sphericity assumption. We preferred this correction to the more conservative Greenhouse-Geisser correction, because in our opinion Type II errors would have been more important than Type I errors in this particular study.

Multiple comparisons were performed between treatments within each time by Tukey HSD test. Student t test for independent groups was also performed to compare treatments within each time, in order to verify if correction for multiple comparisons (included in Tukey test) could have concealed possibly interesting effects. All statistical analyses were planned a priori, with the exception of multiple comparisons, performed on a post-hoc basis.

For all analyses, the BMDP statistical package was used [20].

## Results

The analysis of rat weight increase from arrival to the day of testing showed a general well being of experimental animals, similar among treatment groups (data not shown).

### Experiment A

The experiment was replicated three times. For each replication 48 rats were used. Animals were housed in eight cages, six animals per cage. Cage-mates received the same treatment.

Treatment groups are described in the following. Group 1: *Arnica montana *D4; Group 2: *Apis mellifica *D4; Group 3: *Atropa belladonna *D4; Group 4: *Hamamelis virginiana *D4; Group 5: *Lachesis *D6; Group 6: *Phosphorus *D6; Group 7: sterile physiological solution of 0.9% NaCl (saline); Group 8: indomethacin i.m. (administered 30 minutes before edema induction).

Tables 2, 3, 4, 5 show the overall mean values (± SD) for each remedy. The global means by treatment group were compared using parametric ANOVA, followed by Tukey HSD test (not shown) and Student t test for independent groups. When treatments are significantly different from saline at t test (p < 0.05), the results are printed in bold. ANOVA results are reported at the foot of tables. For all combinations of edema model and administration route, we observed significant main effects of treatment and time and significant interaction treatment × time. This was expected, due to the presence of Indomethacin (standard treatment), which always led to a significant inhibition of inflammation in all the experimental conditions (range of reduction from 14% to 40%).

**Table 2:** Experiment A: Carrageenan edema and oral administration

**Drug**	**Paw volume increase over time (ml ± SD) and effect (% of negative control)**							
	
	**1 hr**		**3 hr**		**5 hr**		**7 hr**	
**Saline**	0.57 ± 0.14		0.98 ± 0.13		0.93 ± 0.14		0.79 ± 0.14	
								
**Arnica D4**	**0.46 ± 0.12**	**-18**	0.98 ± 0.14	0	0.85 ± 0.12	-8	0.72 ± 0.11	-9
**Apis D4**	**0.42 ± 0.12**	**-26**	0.90 ± 0.15	-8	**0.77 ± 0.14**	**-17**	**0.65 ± 0.15**	**-18**
**Atropa D4**	**0.44 ± 0.09**	**-22**	0.94 ± 0.16	-4	**0.80 ± 0.18**	**-14**	0.73 ± 0.17	-7
**Hamamelis D4**	0.49 ± 0.11	-14	**0.88 ± 0.14**	**-10**	**0.78 ± 0.17**	**-16**	**0.68 ± 0.14**	**-14**
**Lachesis D6**	**0.41 ± 0.10**	**-28**	**0.87 ± 0.18**	**-11**	**0.81 ± 0.17**	**-12**	**0.62 ± 0.18**	**-21**
**Phosphorus D6**	**0.41 ± 0.13**	**-28**	**0.86 ± 0.14**	**-12**	**0.78 ± 0.13**	**-16**	**0.65 ± 0.11**	**-18**
								
**Indomethacin i.m.**	**0.38 ± 0.14**	**-34**	**0.63 ± 0.18**	**-36**	**0.59 ± 0.18**	**-36**	**0.49 ± 0.18**	**-38**

ANOVA results. Treatment: F(7,136) = 8.38, p < 0.0001; Time: F(3,408) = 674.23, p < 0.0001; Treatment × time: F(21,408) = 3.65, p < 0.0001.

Bold characters denote significant comparisons (by Student t test) vs. Saline group corresponding for time since edema induction.

**Table 3:** Experiment A: Carrageenan edema and administration by sub plantar injection

**Drug**	**Paw volume increase over time (ml ± SD) and effect (% of negative control)**							
	
	**1 hr**		**3 hr**		**5 hr**		**7 hr**	
**Saline**	0.63 ± 0.09		0.92 ± 0.14		0.84 ± 0.13		0.81 ± 0.14	
								
**Arnica D4**	0.59 ± 0.15	-7	0.90 ± 0.14	-2	0.86 ± 0.15	3	0.74 ± 0.15	-8
**Apis D4**	**0.57 ± 0.08**	**-10**	0.83 ± 0.14	-10	0.80 ± 0.13	-5	**0.67 ± 0.12**	**-17**
**Atropa D4**	0.61 ± 0.09	-4	0.96 ± 0.09	4	0.81 ± 0.10	-3	0.74 ± 0.15	-9
**Hamamelis D4**	0.62 ± 0.13	-3	0.95 ± 0.15	3	0.87 ± 0.17	5	0.69 ± 0.12	-15
**Lachesis D6**	0.64 ± 0.16	1	0.86 ± 0.22	-7	0.85 ± 0.20	2	0.81 ± 0.12	0
**Phosphorus D6**	0.59 ± 0.09	-8	0.94 ± 0.13	2	0.88 ± 0.16	6	0.74 ± 0.13	-9
								
**Indomethacin i.m.**	**0.54 ± 0.12**	**-14**	**0.69 ± 0.12**	**-25**	**0.63 ± 0.11**	**-25**	**0.49 ± 0.05**	**-40**

ANOVA results. Treatment: F(7,86) = 8.55, p < 0.0001; Time: F(3,258) = 221.19, p < 0.0001; Treatment × time: F(21,258) = 3.18, p < 0.0001.

Bold characters denote significant comparisons (by Student t test) vs. Saline group corresponding for time since edema induction.

**Table 4:** Experiment A: Blood edema and oral administration

**Drug**	**Paw volume increase over time (ml ± SD) and effect (% of negative control)**							
	
	**1 hr**		**2 hr**		**3 hr**		**5 hr**	
**Saline**	0.60 ± 0.12		0.63 ± 0.11		0.55 ± 0.15		0.36 ± 0.13	
								
**Arnica D4**	0.58 ± 0.08	-5	0.61 ± 0.08	-4	0.52 ± 0.11	-5	0.38 ± 0.11	7
**Apis D4**	0.55 ± 0.08	-8	0.66 ± 0.08	4	0.55 ± 0.15	0	0.35 ± 0.13	-1
**Atropa D4**	0.57 ± 0.08	-6	0.63 ± 0.11	-1	0.54 ± 0.15	-2	0.33 ± 0.11	-7
**Hamamelis D4**	0.57 ± 0.07	-6	0.62 ± 0.08	-2	0.50 ± 0.13	-9	0.31 ± 0.10	-12
**Lachesis D6**	0.55 ± 0.14	-10	0.63 ± 0.11	-1	0.50 ± 0.15	-9	0.31 ± 0.12	-13
**Phosphorus D6**	0.54 ± 0.08	-11	0.62 ± 0.09	-2	0.53 ± 0.12	-4	0.33 ± 0.09	-7
								
**Indomethacin i.m.**	**0.51 ± 0.10**	**-16**	**0.46 ± 0.10**	**-27**	**0.36 ± 0.12**	**-35**	**0.22 ± 0.10**	**-39**

ANOVA results. Treatment: F(7,135) = 4.40, p = 0.0002; Time: F(3,405) = 535.25, p < 0.0001; Treatment × time (F(21,405) = 2.66, p = 0.0004.

Bold characters denote significant comparisons (by Student t test) vs. Saline group corresponding for time since edema induction.

**Table 5:** Experiment A: Blood edema and administration by sub plantar injection

**Drug**	**Paw volume increase over time (ml ± SD) and effect (% of negative control)**							
	
	**1 hr**		**2 hr**		**3 hr**		**5 hr**	
**Saline**	0.80 ± 0.08		0.73 ± 0.09		0.69 ± 0.08		0.52 ± 0.07	
								
**Arnica D4**	**0.65 ± 0.09**	**-19**	0.68 ± 0.11	-7	**0.60 ± 0.08**	**-12**	**0.47 ± 0.07**	**-9**
**Apis D4**	**0.69 ± 0.09**	**-14**	0.69 ± 0.08	-6	**0.63 ± 0.08**	**-8**	**0.45 ± 0.07**	**-14**
**Atropa D4**	**0.73 ± 0.09**	**-9**	0.70 ± 0.09	-4	**0.63 ± 0.09**	**-8**	0.48 ± 0.08	-9
**Hamamelis D4**	**0.67 ± 0.13**	**-16**	0.70 ± 0.13	-5	0.64 ± 0.09	-7	**0.45 ± 0.09**	**-14**
**Lachesis D6**	0.77 ± 0.09	-4	0.75 ± 0.11	3	0.63 ± 0.09	-8	0.48 ± 0.09	-8
**Phosphorus D6**	**0.63 ± 0.13**	**-21**	0.69 ± 0.16	-5	**0.60 ± 0.15**	**-12**	0.45 ± 0.15	-13
								
**Indomethacin i.m.**	**0.69 ± 0.08**	**-14**	**0.62 ± 0.09**	**-16**	**0.49 ± 0.09**	**-28**	**0.38 ± 0.11**	**-27**

ANOVA results. Treatment: F(7,134) = 3.92, p = 0.0006; Time: F(3,402) = 579.79, p < 0.0001; Treatment × time: F(21,402) = 4.54, p < 0.0001.

Bold characters denote significant comparisons (by Student t test) vs. Saline group corresponding for time since edema induction.

Tables 2 and 3 respectively show the activity of the compounds administered orally or by sub plantar injection on paw volume during carrageenan-induced edema. All the orally administered homeopathic remedies had an inhibitory effect above all one hour after edema induction, particularly *Apis*, *Lachesis *and *Phosphorus *(Table 2). When administered by means of sub plantar injections, the effect of the same homeopathic remedies was significant only in the case of *Apis*, after 1 and 7 hours (Table 3).

In the blood-induced edema model of inflammation none of the orally administered remedies showed a significant effect when compared to saline solution (Table 4).

The homeopathic remedies administered by sub plantar injections (Table 5) led to a slight inhibition of blood-induced edema, particularly 1 hour after the administration of *Arnica*, *Apis*, *Hamamelis *and *Phosphorus*. None showed any significant inhibitory effect after 2 hours; *Arnica *and *Apis *induced a significant inhibition after 3 and 5 hours, *Atropa *and *Phosphorus *after 3 hours and *Hamamelis *after 5 hours.

The results of Student t test are confirmed also by the results of Tukey test (not shown).

### Experiment B

On the basis of the results of Experiment A, in phase B we chose to replicate the carrageenan edema model in rats treated orally with *Apis*, *Lachesis*, *Phosphorus*. The experiment was replicated three times using 48 rats for each replication. Cagemates received different treatments; therefore, no confounding effect of cage could interfere in the assessment of treatment effect.

Treatment groups are described in the following. Group 1: *Apis mellifica *D4; Group 2: *Apis mellifica *D30; Group 3: *Lachesis *D6; Group 4: *Lachesis *D30; Group 5: *Phosphorus *D6; Group 6: *Phosphorus *D30; Group 7: sterile physiological solution of 0.9% NaCl (saline); Group 8: indomethacin i.m. (administered 30 minutes before edema induction).

Differential paw volume data in the various subgroups are presented as mean ± SD (Table 6). The global means by treatment groups were compared using Student t test for independent groups. When significantly different from saline at t test (p < 0.05), the results are printed in bold. ANOVA results are reported at the foot of tables. Also in Experiment B we observed significant main effects of treatment and time and significant interaction treatment × time. Specifically, when compared to saline, indomethacin showed a significantly lower increase in paw volume, while no homeopathic treatment at any dosage gave significant results. When examining separate replications, significant differences between saline and homeopathic treatments (with homeopathic treatments performing worse than saline) were rare and sparse, mainly concentrated at 5 hours from edema induction. Clear-cut effect of dilutions was never observed.

**Table 6:** Experiment B: Carageenan edema and oral administration

**Drug**	**Paw volume increase over time (ml ± SD) and effect (% of negative control)**							
	
	**1 hr**		**3 hr**		**5 hr**		**7 hr**	
**Saline**	0.39 ± 0.17		0.77 ± 0.16		0.63 ± 0.18		0.54 ± 0.16	
								
**Apis D4**	0.37 ± 0.11	-7	0.81 ± 0.11	+6	0.70 ± 0.20	+12	0.60 ± 0.15	+11
**Apis D30**	0.43 ± 0.11	+8	0.82 ± 0.11	+7	0.73 ± 0.17	+16	0.59 ± 0.13	+10
**Lachesis D6**	0.40 ± 0.12	+1	0.79 ± 0.19	+3	0.71 ± 0.18	+14	0.58 ± 0.17	+7
**Lachesis D30**	0.40 ± 0.17	+2	0.79 ± 0.19	+3	0.75 ± 0.19	+19	0.59 ± 0.21	+10
**Phosphorus D6**	0.40 ± 0.15	+1	0.80 ± 0.18	+4	0.70 ± 0.16	+11	0.59 ± 0.20	+10
**Phosphorus D30**	0.41 ± 0.16	+3	0.85 ± 0.23	+11	0.72 ± 0.21	+14	0.59 ± 0.18	+10
								
**Indomethacin i.m.**	**0.30 ± 0.14**	**-24**	**0.48 ± 0.15**	**-38**	**0.52 ± 0.10**	**-18**	**0.43 ± 0.10**	**-20**

ANOVA results for measurements from 1 to 7 hrs since edema induction. Treatment: F(7,14) = 4.23, p = 0.0105; Time: (F(3,6) = 26.91, p = 0.0007; Treatment × time (different profiles over time between treatments): F(21,42) = 2.07, p = 0.1010; Replication × time (different profiles over time between replications): F(6,45) = 8.61, p < 0.0001; Replication × treatment × time (different profiles over time between replications and treatments): F(42,315) = 1.65, p = 0.0110.

Bold characters denote significant comparisons (by Student t test) vs. Saline group corresponding for time since edema induction.

The results of Student t test are confirmed also by the results of Tukey test (not shown).

Finally, nine measures of paw volume were collected on one non-treated rat (arrival weight 150 g, weight at the day of testing 200 g) to estimate the reproducibility of the measurement instrument. The measurements ranged from 1.14 to 1.40, with a mean of 1.25 and a SD of 0.08. The coefficient of variation was therefore CV = 6.09%.

## Discussion

Relatively few pre-clinical studies have been carried out in toxicological research in order to verify the effects of homeopathic remedies under standardized conditions and their results are not incontrovertible [2-17,19,21].

The present study was designed to explore the possibility to test in a controlled way the effects of homeopathic remedies on two known experimental models of acute inflammation in the rat. To this aim, the study considered six different remedies indicated by homeopathic practice for this type of symptom, in two experimental edema models (carrageenan- and autologous blood-induced edema), using two treatment administration routes (sub-plantar injection and oral administration).

On the overall the study involved more than 700 rats in one of the largest pre-clinical study of the effects of homeopathic remedies ever performed.

In the first series of experiments (phase A) some statistically significant effects of homeopathic remedies were observed in two experimental conditions: oral administration in carrageenan-induced edema and sub plantar administration in blood-induced edema (reduction in paw volume increase up to 28% and 21% compared to the saline control, respectively). These effects were more evident and statistically significant in the initial and/or final phases of inflammation, when it is less marked and probably easier to control. The most relevant results concerned *Apis*, *Lachesis *and *Phosporus *in the oral treatment of carrageenan-induced edema (with a range of edema reduction from 11% to 28%). The anti-inflammatory effects of the homeopathic remedies were approximately 50% less than those of the reference drug indomethacin.

When retested in phase B, where a double-blinding procedure and coding of remedies was performed, the three tested homeopathic remedies (at different dilutions) did not show any anti-inflammatory effect. The lack of reproducibility of the results in the second experiment may be explained by the different experimental protocol used. In particular, two experimental conditions were modified in the second phase: the generation of allocation sequences to the treatments and the blinding of these sequences. Moreover, the blinding process could have altered the storage of homeopathic remedies (glass in A, plastic in B), and this was an additional source of difference in the protocols between experiments A and B.

Indeed, in the first experiment animals were randomly allocated to cages and all animals hosted in the same cage received the same treatment. Although it is unlikely that a "cage-effect" has occurred during the first experiment, in the second one, animals were randomly distributed in different cages and in each cage animals received the different treatments at random. It is conceivable, at least in theory, that some unknown "cross-effect" among animals in the same cage, treated with different remedies, could have taken place, thus reducing the (already low) net difference between *verum *and placebo. It is important to underline that the activity of indomethacin was reproduced in all phases of the experiment, suggesting that if some cross-contamination occurred, this would have affected only saline-treated and homeopathy-treated rats reducing all responses and decreasing a possible (small) effect of homeopathic drugs.

Experiment B differs from A also by inclusion of higher potencies (D30), which were hypothesized to "radiate" through sealed ampoules [22]. This elusive effect could leave open the possibility of some cross-contamination during some stages of the experimental process that we have not considered in the protocol.

Furthermore, it is important to consider the possibility of an (unconscious) effect of the researchers, due to the absence of blinding of treatment allocation in the first phase. This may be the case if the induction of edema, the administration of treatments or the collection of the response variable were performed in different ways for animals treated with *verum*, with placebo or with the active reference drug. This confounding effect is rarely controlled in conventional animal research (thus constituting a possible weak point of this discipline), while it is appropriately taken into account almost always in clinical research. However, even if it is difficult to conceive how this confounding effect could have acted in our experimentation, it cannot be totally excluded [23,24]. The fact that the effects of the homeopathic dilutions studied in pre-clinical tests carried out by different research groups, when observed, are often small and difficult to reproduce [11], emphasizes the relevance of the experimental conditions in which significant effects can be observed.

The experimental models we used to evaluate anti-inflammatory treatments have explored what is conventionally denoted as pharmacological "activity" on one symptom (e.g. foot swelling). While conventional anti-inflammatory drugs are designed to suppress the underlying enzymatic mechanism of inflammation (e.g. prostaglandin production), homeopathic treatment is supposed to regulate the pathological excess of inflammation because the phenomenon by itself is seen as an expression of natural healing dynamics (the so called Hahnemann's "life force"). According to classical homeopathic theory, an "anti-edema" effect could not reflect the full potential of the homeopathic treatments of inflammatory diseases. On the other hand, other experimental approaches and/or different formulations showed consistent anti-inflammatory effects of homeopathic remedies such as *Arnica compositum *[2,3], *Apis *[7] and *Arnica Montana *[25] using the rat-paw edema model. So, other technical factors, such as the composition of the medicines (e.g. single remedies versus complex formulations), and the type of solvent used (water, saline, water/alcohol mixtures) [26], may explain the observed discrepancies. If a homeopathic treatment acts by influencing the natural healing dynamics of the whole treated subject by means of small doses or highly diluted administrations, this action could be, at least in theory, highly sensitive to even small changes in experimental conditions [27]. Moreover, when used in humans, a homeopathic treatment is also chosen on the basis of the global pathophysiological characteristics of the individual, and not only in relation to local symptoms.

## Conclusion

In conclusion, the discrepancies observed in the two phases of our study make it possible to draw some suggestions useful for designing possible further experiments: a) the effects, when obtained, are relatively small, thus experimental conditions which may affect the response, increasing the variability between experiment results, should be accurately controlled; b) the discrepancies between single-blind (measurement) and double-blind (treatment administration and measurement) methods in animal pharmacological research are noteworthy and suggest that full blinding of procedures (drug administration, data collection and analysis) may be a critical factor for the results of animal experimental investigations, also in non-homeopathic research.

## Competing interests

The author(s) declare that they have no competing interests.

## Authors' contributions

AC, PB, SB conceived the study, participated in its design and carried out the experiment. FC and FMI, refined the experimental design, provided the power calculation, performed the statistical analyses and drafted the manuscript. RR, participated in the study design, coordinated the study and drafted the manuscript. All authors read and approved the final manuscript.

## Pre-publication history

The pre-publication history for this paper can be accessed here:


